# Hysterectomy—Current Methods and Alternatives for Benign Indications

**DOI:** 10.1155/2010/356740

**Published:** 2010-07-28

**Authors:** Michail S. Papadopoulos, Athanasios C. Tolikas, Dimosthenis E. Miliaras

**Affiliations:** Department of Obstetrics and Gynecology, Euromedica General Clinic of Thessaloniki, Gravias 2, 54645, Thessaloniki, Greece

## Abstract

Hysterectomy is the commonest gynecologic operation performed not only for malignant disease but also for many benign conditions such as fibroids, endometrial hyperplasia, adenomyosis, uterine prolapse, dysfunctional uterine bleeding, and cervical intraepithelial neoplasia. There are many approaches to hysterectomy for benign disease: abdominal hysterectomy, vaginal hysterectomy, laparoscopic assisted vaginal hysterectomy (LAVH) where a vaginal hysterectomy is assisted by laparoscopic procedures that do not include uterine artery ligation, total laparoscopic hysterectomy (TLH) where the laparoscopic procedures include uterine artery ligation, and subtotal laparoscopic hysterectomy (STLH) where there is no vaginal component and the uterine body is removed using a morcelator. In the last decades, many new techniques, alternative to hysterectomy with conservation of the uterus have been developed. They use modern technologies and their results are promising and in many cases comparable with hysterectomy. This paper is a review of all the existing hysterectomy techniques and the alternative methods for benign indications.

## 1. Introduction

The term hysterectomy originates from two Greek words: “hystero” which means uterus and “ectomy” which means resection removal from the human body. This surgical procedure is indicated in several common gynecologic problems. Hysterectomy is either total or subtotal, with or without the adnexae and depended on the way performed: abdominal, vaginal and laparoscopic or laparoscopic assisted vaginal hysterectomy. Historically the first vaginal hysterectomy was performed by Conrad Langenbeck in 1813, the first subtotal abdominal hysterectomy by Walter Burnham in 1853, the first elective abdominal hysterectomy by Clay and Koeberle in 1863, and the first laparoscopic hysterectomy by Harry Reich in 1988.

## 2. Material and Methods

There are several indications for hysterectomy or the alternative procedures with preservation of the uterus which will be analyzed in detail. In this paper, only benign conditions will be reviewed. 

### 2.1. Fibroid Uterus

Fibroids (myomas) originate from the uterine smooth muscle wall. The percentage of malignant transformation to sarcoma is 0,1–0,8% according to various references. Fibroids, depending on size and location can cause menorrhagia or symptoms from pressure to adjacent organs, for example, ureters, bladder, or intestine. Before proceeding to hysterectomy for a fibroid uterus, reproductive activity of the patient must be completed. 

#### 2.1.1. Surgical Approach

**(I) Abdominal Hysterectomy:**

- *Total*: with or without bilateral salpingoophorectomy.
- *Subtotal*: with or without bilateral salpingoophorectomy.

**(II) Laparoscopic Hysterectomy:**

- *Total*: (+/− initially laparoscopic myomectomy for reduction of the uterine volume): with or without bilateral salpingoophorectomy.
- *Subtotal*: with or without bilateral salpingoophorectomy.

**(III) Laparoscopic Assisted (Total) Vaginal Hysterectomy (LAVH)**

(+/− initially laparoscopic myomectomy for reduction of the uterine volume): with or without bilateral salpingoophorectomy.

**(IV) Vaginal (Total) Hysterectomy:**

with or without bilateral salpingoophorectomy.

#### 2.1.2. Alternative Methods

- Myomectomy:
  - via laparotomy,
  - via laparoscopy.
- Uterine artery embolism (UAE).
- Transvaginal Temporary Uterine Artery Occlusion.
- MRI-Guided Focused Ultrasound (MRgFUS).
- Medical treatment (progesterone mifepristone and asoprisnil under investigation).

### 2.2. Endometrial Hyperplasia

Endometrial hyperplasia with atypia associated with a high risk for malignant transformation, is an absolute indication for hysterectomy. In endometrial hyperplasia without atypia, hysterectomy is indicated only if pharmacological agents cannot control menorrhagia. 

 There are four categories of endometrial hyperplasia:

- simple hyperplasia without atypia,
- complex hyperplasia without atypia,
- simple hyperplasia with atypia,
- complex hyperplasia with atypia.

#### 2.2.1. Surgical Approach

**(I) Abdominal Hysterectomy:**

- *Total*: with or without bilateral salpingoophorectomy.
- *Subtotal*: with or without bilateral salpingoophorectomy.

**(II) Laparoscopic Hysterectomy:**

- *Total*: with or without bilateral salpingoophorectomy.
- *Subtotal*: with or without bilateral salpingoophorectomy.

**(III) Laparoscopically Assisted (Total) Vaginal Hysterectomy (LAVH):**

with or without bilateral salpingoophorectomy.

**(IV) Vaginal (Total) Hysterectomy:**

with or without bilateral salpingoophorectomy.

#### 2.2.2. Alternative Methods

Operative hysteroscopy and resection of the endometrium (endometrectomy) using a resectoscope in case of hyperplasia without atypia (Trans Cervical Resection of the Endometrium—TCRE), 

 Operative hysteroscopy and cauterization of the endometrium using a resectoscope in case of hyperplasia without atypia (Rollerball), 

 Thermal uterine balloon therapy system for endometrial ablation in case of hyperplasia without atypia, 

 Medical treatment (pharmacological agents such as progesterone) in case of hyperplasia without atypia, 

 Insertion of levonorgestrel hormone releasing intrauterine device (LNG-IUD) in case of hyperplasia without atypia.

### 2.3. Adenomyosis

A benign condition of the uterus which can cause menorrhagia characterized by diffuse spread of ectopic endometrium in the myometrium. Hysterectomy is indicated when other therapeutic approaches have failed to control symptoms. 

#### 2.3.1. Surgical Approach

**(I) Abdominal Hysterectomy:**

- *Total*: with or without bilateral salpingoophorectomy.
- *Subtotal*: with or without bilateral salpingoophorectomy.

**(II) Laparoscopic Hysterectomy:**

- *Total*: with or without bilateral salpingoophorectomy.
- *Subtotal*: with or without bilateral salpingoophorectomy.

**(III) Laparoscopic Assisted (Total) Vaginal Hysterectomy (LAVH):**

with or without bilateral salpingoophorectomy.

**(IV) Vaginal (Total) Hysterectomy:**

with or without bilateral salpingoophorectomy.

#### 2.3.2. Alternative Methods

Adenomyomatectomy via laparotomy or laparoscopy. 

Combination of hysteroscopic resection of the endometrium (Trans Cervical Resection of the Endometrium—TCRE) and hysteroscopic cauterization to the endometrium with the Rollerball device.

### 2.4. Uterine Prolapse

It is not an absolute indication for hysterectomy. In case of absence of other uterine pathology (such as sarcoma, carcinoma of the endometrium, carcinoma of the cervix) the translocation of the uterus in its natural position and the surgical fixation of the uterus in the pelvis have better results than hysterectomy in the anatomy of the pelvic floor. 

#### 2.4.1. Surgical Approach

**(I) Total Hysterectomy:**

- *Abdominal Total*: with or without bilateral salpingoophorectomy.
- *Vaginal Total*: with or without bilateral salpingoophorectomy.
- *Laparoscopic Total*: with or without bilateral salpingoophorectomy and sacrocolpopexy or lateral fixation of the vaginal vault using the J.B. Dubuisson method (using a polypropylene mesh). Laparoscopic approach is accompanied with a “Burch” or a “free Tension Vaginal Tape” procedure.
- *Laparoscopic assisted total vaginal hysterectomy*: with or without bilateral salpingoophorectomy.

**(II) Subtotal Hysterectomy:**

Only in Cases That Cervical Pathology, for Example, CIN or Cervical Carcinoma Has Been Excluded

- via laparotomy + cervicosacropexy,
- via laparoscopy + cervicosacropexy or lateral fixation of the cervical stump using the J.B. Dubuison method (using a polypropylene mesh).

#### 2.4.2. Alternative Methods

**Conservation of the Uterus:**

When Other Uterine Pathology Has Been Excluded and the Only Problem Is the Prolapse.

- Fixation of the uterine ligaments by laparotomy or laparoscopy.
- Fothergill isthmical uteropexy (Manchester repair) in case of simple elongation of the cervix.
- Isthmical sacropexy (sacrocolpopexy) by laparotomy or laparoscopy.
- Kapandji procedure.
- Colpoclisis.

### 2.5. Dysfunctional Uterine Bleeding (DUB)

This common cause of abnormal uterine bleeding pattern is diagnosed when anatomical causes have been excluded. The problem is a hormonal imbalance in the hypothalamus-hypophysis-gonads axis. 

#### 2.5.1. Surgical Approach

**(I) Abdominal Hysterectomy:**

- *Total*: with or without bilateral salpingoophorectomy.
- *Subtotal*: with or without bilateral salpingoophorectomy.

**(II) Laparoscopic Hysterectomy:**

- *Total*: with or without bilateral salpingoophorectomy.
- *Subtotal*: with or without bilateral salpingoophorectomy.

**(III) Laparoscopic Assisted (Total) Vaginal Hysterectomy (LAVH):**

with or without bilateral salpingoophorectomy.

**(IV) Vaginal (Total) Hysterectomy:**

with or without bilateral salpingoophorectomy.

#### 2.5.2. Alternative Methods

- Insertion of levonorgestrel hormone-releasing intrauterine device (LNG-IUD).
- Rollerball electrocoagulation (RBE).
- Nd:YAG laser ablation.
- Transcervical resection of endometrium (TCRE).
- Thermal uterine balloon therapy system for endometrial ablation.
- Global 3D Bipolar Ablation Method.
- Punctual Vaporizing Method.
- Endometrial Ablation by Intrauterine Instillation of Hot Saline.
- Diode Laser Method.
- Photodynamic Therapy.
- Microwave Method.
- Radiofrequency Method.
- Cryotherapy Method.

### 2.6. High Grade Cervical Intraepithelial Neoplasia (CIN III)

In younger patients who have not completed their family an excision of the transformation zone using electrocautery (LLETZ) or a classical conisation of the cervix using scalpel or laser is indicated. In older patients who have completed their family, trachelectomy or even an abdominal hysterectomy with or without the ovaries is indicated.

A review of all the existing and especially the new techniques of hysterectomy and its alternatives follows.

1. Total laparoscopic hysterectomy (TLH) with or without salpingoophorectomy.
2. Subtotal laparoscopic hysterectomy (STLH) with or without salpingoophorectomy.
3. Laparoscopic assisted vaginal hysterectomy (LAVH) with or without salpingoophorectomy.
4. Laparoscopic adenomyomectomy in case of focal adenomyosis.
5. Isthmical sacropexy (sacrocolpopexy with conservation of the uterus).
6. Cervicosacropexy after laparoscopic subtotal hysterectomy.
7. Lateral fixation of the cervical stump or the vaginal vault (J.B. Dubuisson method).
8. Uterine artery embolism (UAE) in case of uterine fibroids.
9. Ttansvaginal temporary uterine artery occlusion.
10. MRI guided focused ultrasound (MRgFUS).
11. Hysteroscopic (transcervical) resection of the endometrium (endometrectomy) with resectoscope (TCRE).
12. Hysteroscopic cautery to the endometrium with resectoscope (Rollerball).
13. Nd-YAG Laser Ablation.
14. Thermal uterine balloon therapy system for endometrial ablation.
15. Global 3D Bipolar Ablation Method.
16. Punctual Vaporizing Method.

#### 2.6.1. Total Laparoscopic Hysterectomy (TLH)

**Position**

The patient is placed in the supine position with both hands on her sides using extension tubes for intravenous access. Her knees slightly bended to avoid pressure. Calves slightly turned laterally. Rumps 10 cm outside the operation table edges. The height of the operation table 30 cm lower than in classical laparotomy. Upper limbs insulated from the operating table.

**Procedure**

There are placement of the uterine manipulator device. Small incision vertical or round on the lower edge of the umbilicus 10 mm in length. Insertion of the Veress needle. Security test using a syringe. Inflation of the peritoneal cavity through the veress needle with CO_2_. The pressure limit is 14–18 mmHg. Insertion of the main trocard 10 mm wide and through that insertion of the laparoscope and inspection of the whole peritoneal cavity. There are placement of the patient in trendelenbourg position and insertion of the lateral trocards (5 mm width) trying to avoid the epigastric vessels (superficial and deep) and umbilical arteries. Placement of the middle operating trocard (5 mm width) above the pubis symphysis. The left round ligament is grasped and uterus is turned onto the other side in order to put tension on it. The round ligament is coagulated using bipolar diathermy and cut using scissors. The parametrium is opened and the frontal sheath is cut to the uterine-urinary bladder peritoneal fold. A “window” is opened on the posterior sheath of the broad ligament. The same procedure on the right side. Ovarian ligaments or suspensor ligaments (depending on the indication) are grasped, coagulated and cut. The uterine-urinary bladder peritoneal fold is grasped, divided and cut to the lateral edge. The frontal vaginal vault then appears. The ascending and descending branches of the uterine arteries are found in the parametrium, coagulated with bipolar diathermy and cut. Uterosacral ligaments are grasped and cut where they conjoint. Vaginal vault is presented using the manipulator and a circular incision is performed and uterus can now be removed through the vagina. Uterus is placed in the vagina so that inflation of the abdomen is not disturbed. Vaginal vault is coagulated only mildly in bleeding spots to avoid tissue necrosis. Suturing of the vaginal vault edges and uterosacral ligaments (2 stitches in the ankles). Checking of the haemostasis. Removal of the instruments and the pneumoperitoneum. Suturing of the wounds where trocards were placed.

#### 2.6.2. Subtotal Laparoscopic Hysterectomy (STLH)

The differences between this procedure and Laparoscopic total hysterectomy are listed below. (1) When parametrium is divided we stop at the isthmus where uterine artery is divided in her ascending and descending branches. Thermal ligation is performed only for the ascending branches. (2) Circular incision is performed round the isthmus and not around the vaginal vault. (3) Uterine body is removed using a morcelator through the middle operative trocard and not through the vagina as in total hysterectomy. (4) Ovaries are placed in a laparoscopic bag when removed. (5) Uterosacral ligaments are not incised. (6) Suturing of the cervical stump and not of the vaginal vault is performed.

#### 2.6.3. Laparoscopic Assisted Vaginal Hysterectomy (LAVH)

In this procedure, laparoscopic approach is used only for the ligation and incision of the round ligaments and the suspensory or ovarian ligaments whether ovaries are going to be removed or not. Ligation and incision of the uterine arteries and the rest of the procedure is performed through the vaginal route.

#### 2.6.4. Laparoscopic Adenomyomectomy (Focal Adenomyosis)

Excision of adenomyomas can be performed using the laparoscopic approach but is completely different when compared to the laparoscopic myomectomy. Surgical procedure is certainly more difficult because adenomyomas have no clear margins from the normal uterine smooth muscle wall. In certain occasions the surgeon is obliged to perform a tumor reduction operation and remove only part of the mass. Another difference comparing with myomectomy is the tissue deficit that the surgeon has to deal with when adenomyoma is removed and the deficit has to be sutured. There are very few references in the international literature but the results of this operation seem promising even when it is performed as a mass-reduction operation.

#### 2.6.5. Laparoscopic Isthmical Sacropexy (Sacrocolpopexy with Conservation of the Uterus)

There are two different techniques. The first is described as lateral fixation only of the right side due to the presence of rectum and sigmoid colon on the left side. The second referred as “Scali” technique is described as bilateral fixation using a “V” shaped mesh. This technique is described below.

**There Are Three Basic Principles**

(a) Suspension of the uterus in its normal position and fixation of the pelvic floor below the urinary bladder. (b) Treatment of the stress incontinence even if the patient is asymptomatic. The reason is that suspension of the uterus will reveal or worsen the stress incontinence. (c) Surgical reconstruction of the rectovaginal diaphragm.

**Surgical Procedure**

There is incision on the peritoneum 1 cm below the urinary bladder—uterine peritoneal fold. The space between the bladder and uterus is exposed and the bladder is pulled downwards. These space's lateral margins (pillars) are joined inferiorly in the height of the posterior urethra. The vaginal wall is exposed and the broad ligament is opened at the isthmus far away from the uterine arteries. A wide window is opened in the posterior fold of the broad ligament. The peritoneum between the uterosacral ligaments is opened to 1 cm above the point the two uterosacral ligaments joint together (“Λ” point). The sacral promontory (L5-S1 space), the iliac veins and the ureters are recognized. The peritoneum covering the sacrum is opened and anterior elongated ligament and middle sacral vessels are recognized. The latest are coagulated only if they cannot be removed from the points that stitches are to be placed. Opening of the peritoneum continues to the point that uterosacral ligaments joint. A “V” shaped mesh is inserted through the middle operating trocard (10–12 mm). The wide part of the mess is placed in the space in front of the urinary bladder and the sides are placed through the windows in the broad ligaments. The sides of the mess are barried behind the uterus at the isthmus. The two pararectal spaces are opened, suspensory muscles of the rectum are recognized and the sides of the mesh are fixed in this space and the Cardinal ligaments. The mesh is sutured on the vaginal wall using 2/0 nonabsorbable stitches with a 23 mm needle (6–8 stitches). Restoration of the peritoneum only in the rectovaginal space follows. Then sacropexy is performed. The two sides of the mesh are fixed on the anterior longitudinal ligament of the spine with two stitches using no1 nonabsorbable stitches with a 30 mm needle. Special attention is needed to avoid injury of the intervertebral disk. Restoration of the peritoneum is completed in order to cover the mesh in the retroperitoneal space.

After completion of the procedure, a Burch colposuspension or a tension-free vaginal tape application is performed for the reasons already mentioned. In case stress incontinence is due to sphincter failure Burch colposuspension is not indicated and a tension-free vaginal tape has to be applied. At the end a typical posterior repair is performed.

**Complications:**

- When opening the space in front of the urinary bladder, injuries to the bladder are common. Injuries to the ureters are more uncommon.
- When opening the broad ligaments, the sigmoid colon can be injured.
- When opening the recto-uterine space, the rectum can be injured.
- When the anterior longitudinal ligament is prepared, bowel, ureters, middle sacral artery, and left iliac vein can be injured.
- Infection of the mesh.

#### 2.6.6. Cervicosacropexy after Laparoscopic Subtotal Hysterectomy

During the time of uterus removal from peritoneal cavity, the mesh that is going to be used (polypropylene Type 1) is drawn and cut out. It is inserted through the medial operating trocard and detained on the cervical stump with staplers and four sutures using nonabsorbent stitch 1, with 23 mm needle. 

 Opening of the peritoneum in front of the anterior elongated ligament and opening of a “channel” between this and the cervical stump. The mesh inserted within the channel and fixation on the anterior elongated ligament with staplers and two sutures using nonabsorbent stitch. Restoration of the peritoneum follows. 

 After completion of the operation, Burch colposuspension or insertion of a tension-free vaginal tape is needed. In cases that urine incontinence is due to sphincter deficiency, Burch procedure has no place and the placement of a tension-free vaginal tape would be preferable. Finally, a typical posterior repair is performed.

#### 2.6.7. Laparoscopic Lateral Fixation with the Use of Synthetic Mesh (J.B. Dubuisson Method)

**(A) Of Cervical Stump (After Laparoscopic Subtotal Hysterectomy)**

During the time of uterus removal from the peritoneal cavity, the mesh that is going to be used (polypropylene Type 1) is drawn and cut out in two elongated parts. These two parts are inserted in the peritoneal cavity through the suprapubic operative trocard and are expanded on the cervical stump. Fixation on the cervical stump using stapler and four nonabsorbent stitches 1, with 23 mm needle follows. A needle holder is inserted over the iliac promontory on the left, two to three centimeters outside the side trocard. Forward this up to the parietal peritoneum. Forward outside the peritoneum until meeting the one side of the mesh. The side of the mesh is grasped and pulled up to the skin and finally fixed with a small grasper. The same surgical procedure to the other side. Restoration of the peritoneum over the cervical stump. Pulling of the sides of the plexus- fixing on the sheath of the external oblique abdominal muscle with nonabsorbent stitches.

Similarly to the classical sacropexy, a Burch colposuspension or insertion of a tension-free vaginal tape is needed afterwards. If incontinence is due to sphincter deficiency, Burch operation has no use and the placement of a tension-free tape would be preferable. Finally, a typical posterior repair suture is performed.

**(B) Of Vaginal Stump (After Laparoscopic Total Hysterectomy)**

It is the same surgical technique. The only difference is that the mesh is fixed on the vaginal and not on the cervical stump.

#### 2.6.8. Uterine Artery Embolization (UAE)

It is a transcutaneous, X-Ray guided technique, performed by specialists in intervention-radiography [1]. An angiography catheter is inserted in the uterine arteries through one of the two common femoral arteries. Infusion of the embolic agents (polyvinyl alcohol particles or tris-acryl gelatin microspheres) follows in the two uterine arteries until their blood flow decelerates [1–7]. 

 The mechanism of UAE is the irreversible ischemic necrosis of the fibromyomas caused by the crucial decrease of the blood flow, having as a result their necrosis, while the rest of the normal myometrium is capable of surviving [8, 9]. The whole procedure takes place under intravenous anesthesia and lasts approximately one hour. 

The recovery is brief and relatively mild, with 4-5 days of recurrent uterine cramping and constitutional symptoms (nausea malaise, fatigue and low-grade fever). Patients can usually return to normal activities within 8–14 days [6, 10–13]. 

UAE technique was first described in 1995 [2]. Several studies show a 50–60% decrease in fibroids dimensions and also a decrease in menorrhagia and the rest of the symptoms related to them in a percentage that reaches 85–95% in short- and middle-term follow-up [3, 5–7, 10, 14–20].These studies also report 87–97% patient satisfaction after the procedure. With concern to the mild complications, according the perspective study FIBROID registry, a percentage of 2,7% (90 out of 3041 patients) in the immediate postoperative period and 26% (710 out of 2729 patients) after the hospital exit are reported. The percentages of the severe complications are 0,6% and 4,1%, respectively, [13]. 

 A common complication of UAE is the infracted fibroid to become endocavitary and “aborted” through the vagina (approximately in 10% of the cases) [6, 21]. According to Fred Burbank [22], this is not a complication, if the patient is well informed and ready to consider this as part of the procedure. In any case, this is usually reported after UAE for submucosal fibroids or fibroids lying within the uterine wall and protruding inside the uterine cavity. According to the literature the “abortion” of such fibroids can take place within 6 months and 4 years [23]. Most of the times the “abortion” is spontaneous while in some cases cervical dilation or even hysteroscopic removal is needed when the fibroid remains connected to the uterus. 

 The most serious complication of UAE is endometritis (less than 1%) [3, 6, 10, 11, 13, 24], which if left without antimicrobial prophylaxis could lead to sepsis, or even death (~0,05/1000) [25]. 

 Concerning the failure rate of the procedure, according to the FIBROID registry study after three years of follow-up there was a need for hysterectomy, myomectomy or a repeat UAE procedure in 9,8%, 2,8% and 1,8%, respectively, [26]. The need for a repeat operation is not only due to new fibroids but also due to incomplete infarction of the old ones [27]. The successful embolization of only one uterine artery is considered as a technical failure of the procedure, since in most of the cases the blood flow to the fibroids derives from both the uterine arteries [28]. There is also dispute among several studies whether the size or the number of fibroids are predictive factors of possible failure [29]. 

Uterine artery embolization (UAE) for fibroids has been extensively investigated. Since 1995 when was first introduced and particularly in the last 5 years, several high-quality studies reported on its outcome have been completed. These studies have demonstrated that when successful, UAE can provide symptom control similar to surgery. Although hysterectomy remains more effective in symptom control and durability, many women are seeking uterine-sparing alternatives. UAE has emerged as the leading minimally invasive treatment for fibroids: Morbidity is low and recovery rapid; serious complications are quite rare. With a few anatomical exceptions, UAE is appropriate for most patients with symptomatic fibroids who have completed childbearing [30]. Although pregnancy is certainly possible after embolization [30–32] existing data suggest better reproductive outcomes for myomectomy in the first 2 years after treatment. The current recommendation is for myomectomy as a first choice for patients seeking to become pregnant.

#### 2.6.9. Transvaginal Temporary Uterine Artery Occlusion

It is an alternative method targeting to decrease the blood flow in the uterine arteries in order to treat fibroids. The theory supporting this technique is that a fibroid necrosis can take place after temporary occlusion of uterine arteries and temporary uterine ischemia that follows [33, 34]. It demands special equipment and the duration of the procedure is 6 hours. Immediately after the procedure the fibroids and the uterine volume decreases 40-50%. In 6 months follow-up, the symptoms improved 80–90%. 

 Comparing transvaginal temporary uterine artery occlusion with uterine artery embolization, in this technique there is no exposure to radiation and the patients has much less postoperative pain. Although the short-term results seem to be similar to UAE, the long-term results are insufficient since the ischemia is much weaker compared to embolization [29]. 

#### 2.6.10. MRI Guided Focused Ultrasound (MRgFUS)

It is a new, minimal interventional method of thermal destruction of fibroids, approved by the US Food and Drug Administration (FDA) since 2004. High frequency ultrasound waves penetrate the anterior abdominal wall and focus in a specific target inside the fibroid increasing the temperature up to 55–90°C, which leads to necrosis (coagulative necrosis) within a few seconds. The simultaneous use of MRI allows the exact focus on the target and real-time temperature feedback [35–37]. The technique demands special equipment, has to be performed by an expertised specialist and under intravenous anesthesia [29]. 

The method is based on cconsecutive exposures to focused sonications (ultrasound energy), lasting 20 seconds each and resulting in a small (0.5 cm^3^) bean-shaped ablated volume. There is a pause of 90 s to elapse for the tissue to return to its baseline temperature, between sonications. Multiple sonications are required to cover the entire target volume, which is typically limited to a maximum of 150 cm^3^ of tissue, and total procedure time is usually over 3 hours. During the procedure short-term lower abdominal pain, leg pain and buttock pain are common. Patients are usually discharged home 1 hour after the procedure and return to usual activities, on average, within 48 hours [35]. 

 After taking into account that the total number of patients treated with MRgFUS is very small worldwide, its assessment and comparison with other techniques are unsafe. Although compared to UAE there is no exposure to radiation and the postoperative pain is less, in MRgFUS there are constrictions concerning the volume and the number of fibroids. There is a relation between time and total volume of fibroids. This technique also has minor results concerning the decrease of the uterus and fibroid volume [29]. The existence of some special characteristics, like fibroids neighboring blood vessels and nerves or sensitive organs like bowel or urinary bladder, diminishes the number of candidates for the method [36]. Preservation of fertility after MRgFUS is not well documented. It is interesting that the procedure is more successful when a three months treatment with GnRH-agonists precedes, so as for the fibroid blood flow to be decreased [38]. 

#### 2.6.11. Hysteroscopic (Transcervical) Resection of the Endometrium (Endometrectomy) with Resectoscope (Transcervical Resection of the Endometrium-TCRE)

In about 20–25% of hysterectomies the indication is menorrhagia without any obvious pathology. TCRE was first described in 1983 from DeCherney and Polan [39], and is still considered as the gold standard alternative method since it combines histological diagnosis and effective treatment. Its target is the resection of the whole endometrium, including basic layer. It is suitable for patients who do not respond to medical treatment, do not want hysterectomy and are not in reproductive age. Before the procedure, malignant disease and atypia in cases of endometrial hyperplasia should be excluded. TCRE is combined with the use of rollerball for the destruction of the endometrium in special spots like fundus and areas around tubal ostia. Preoperative treatment with GnRH-agonists or danazol is not necessary. Postoperatively, the placement of levonorgestrel hormone-releasing intrauterine device (LNG-IUD) has been proposed. Especially in adenomyosis cases this combination increases the success rate of the procedure. In a brief description, endometrium resection starts from the fundus to the isthmus of the uterus clockwise. The resection ends to the isthmus because of the risk of subsequent haematometra. As already mentioned the fundus is coagulated using rollerball along with the tubal ostia and the whole area of endometrial resection [40].

#### 2.6.12. Hysteroscopic Cauterization of the Endometrium with Resectoscope (Rollerball)

**First Generation Endometrial Ablation Method**

The technique was first described by Vancaille in 1989 [41] and as TCRE, is among the most effective first generation methods, done under hysteroscopic guidance. It is important that the endometrium should be as thin as possible, when applied. That is why follicular phase of the menstrual cycle is preferred. The results are even better if GnRH- agonists or danazole are administered for one or two cycles before the operation [42]. The success rate (amenorrhea) after the procedure reaches 100% against 75% without any previous pharmaceutical treatment, the operative time is less because of better visibility, the patients safety and the surgeons effortlessness much greater. It is suitable for these patients who do not respond to pharmaceutical treatment, do not want hysterectomy and are not in reproductive age. Before the procedure malignancy and atypia should be excluded. The technique uses monopolar energy at 60–80 Watt in order to destroy the endometrium through coagulation or at 160 watt to destroy it through vaporization. The percentage of the remnant functional endometrium rates at 70% [43]. So it would be favorable for the patient to administer a combination of estrogen-progesterone or to place a levonorgestrel hormone-releasing intrauterine device (LNG-IUD).

It seems that the combination of the two methods (TCRE-ROLLERBALL) is the gold standard when concerning the endometrium destruction and the avoidance of hysterectomy [40]. Mistletoe study which included 16087 patients in the United Kingdom, showed less complications with the usage of rollerball in difficult to reach with the loop spots, like fundus and the tubal ostia. The same study showed that 75–85% of the patients were very satisfied [44, 45].

#### 2.6.13. Nd-YAG Laser Ablation

**First Generation Endometrial Ablation Method**

It was introduced in 1981 by Goldrath et al. [46]. It requires preoperative hormonal treatment to assure that the endometrium is thin and atrophic. During the procedure there is no use of electric energy and Normal Saline or Dextrose 5% in 50% Saline or Ringer's Lactate Solution is used to dilate the endometrial cavity. There are three techniques: dragging or touching (touch technique), blanching (nontouch technique) and dragging and blanching in combination (combination technique). There is no bleeding because the treatment is strictly by coagulation. In order to avoid meeting the same areas twice there is need for strict charting of the endometrial cavity. The most common technique used is the combination technique; nontouch technique is used in the cornea areas and touch technique in the rest of the endometrial cavity. The disadvantage of Nd-YAG Laser ablation is that it is expensive because of the need for single-use optical fibers [40].

#### 2.6.14. Thermal Uterine Balloon Therapy System for Endometrial Ablation

**Second Generation Endometrial Ablation Method**

There are many companies manufacturing such systems of thermal endometrial destruction. The technique uses the successful heating of some dilative liquid within the balloon that is placed inside the endometrial cavity. The procedure lasts about 8 to 15 minutes depending on the manufacturing company and the success rate of endometrial destruction varies in different studies. The temperature of the liquid inside the balloon ranges from 75–80°C to 87 + 5°C depending again on the manufacturer. Although the procedure takes place without hysteroscopic guidance and is characterized as “blind”, this second generation technique is safe because the strict observation of all the crucial parameters for the patient's safety. Before the procedure the existence of endometrial polyps or fibroids, congenital anomalies of the uterus, pregnancy, pelvis inflammation, endometrium hyperplasia with atypia and malignancies of the endometrium or the cervix should be excluded. This method should take place during the follicular phase of the menstrual cycle [40].

#### 2.6.15. Global 3D Bipolar Ablation Method

**Second Generation Endometrial Ablation Method**

In this technique a three-dimension double polar device is used. The device is connected to a RF generator and causes ablation by coagulation of the endometrium and the superjacent myometrium in a certain set depth. The diameter of the device is 6,5 mm. The power of the generator is fixed to 180 W. It is completed in about one minute and does not demand previous hormonal treatment. The success rate (amenorrhea) is about 80% [40].

#### 2.6.16. Punctual Vaporizing Method

**Second Generation Endometrial Ablation Method**

This technique uses vaporization and not coagulation of the endometrium. Two different devices are manufactured by two different companies. The first uses monopolar energy, the second bipolar. These devices can perform desiccation, vaporization or blended cut depending on the generator adjustment. Their use is not only restricted to endometrial destruction, but can be used in the treatment of submucosal fibroids, polyps and adhesiolysis. The advantage of the bipolar device is the use of Normal Saline for dilatation which makes it safer even in the procedure lasts longer [40].

Other Second-Generation Endometrial Ablation Methods are: Endometrial Ablation by Intrauterine Instillation of Hot Saline, Diode Laser Method, Photodynamic Therapy, Microwave Method, Radiofrequency Method, and Cryotherapy Method.

## 3. Discussion

Independently the technique used, hysterectomy is one of the most common gynecological procedures. There are certain differences comparing various techniques related to indications, advantages and disadvantages. Abdominal hysterectomy, the most well established method, permits the surgeon to deal with any kind of pathology malignancy included, and has the benefit of the direct touch on the tissues. It also offers the benefit of the direct three-dimension visualization of the surgical field and additionally does not warrant expensive special instruments. On the other hand, laparoscopic approach is not indicated for malignant disease due to the hazard of spreading malignant cells by the gas (CO2) used to inflate the abdominal cavity, there is no direct touch on the tissues and warrants specialized surgeons and expensive instruments and equipments. Visualization is in two dimensions which require familiarization with the technique. Despite the disadvantages, laparoscopic approach offers better view of the whole abdominal cavity, magnification, enhanced ability to perform delicate manipulation of the tissues, blood loss is minimal and recovery of the patient is quicker with thrombotic complications occurring less often. In addition post laparoscopic procedure adhesions are rare compared to laparotomy procedures, pain is less, restoration of the gastrointestinal tract function is quicker, scarring much less and duration of in-hospital stay shorter. Another advantage of laparoscopy is the ability to record the procedure. 

 There are certain differences comparing total laparoscopic hysterectomy and laparoscopic assisted vaginal hysterectomy. In the first, vaginal removal of the uterus follows complete laparoscopic ligation and excision of all the pedicles. In the second, ligation and excision of the uterine arteries and the rest of the pedicles is performed using the vaginal route. In more details, in total laparoscopic hysterectomy thermal ligation and excision of the upper and the lower brands of the uterine arteries is performed, in subtotal laparoscopic hysterectomy thermal ligation and excision only of the upper brands of the uterine arteries and in laparoscopic assisted vaginal hysterectomy ligation and excision of the main uterine arteries. Vaginal hysterectomy on the other hand when indicated is a procedure with excellent results. 

 The alternative to hysterectomy techniques, which became available recently and most of them used advanced technology have also very good results when compared to hysterectomy. Most of them are indicated in case of menorrhagia and are based on endometrial ablation. Even if the control of blood loss is not 100% as it is in hysterectomy, it is satisfactory. In cases of menorrhagia due to fibroids the alternative methods are based in the shrinkage of them by tissue necrosis or disturbance to their blood supply. The results in this case are less satisfactory when compared to hysterectomy or myomectomy. Finally when uterine prolapse is the indication for hysterectomy, the results of the alternative techniques are comparable and even better than hysterectomy. The surgical removal of a uterus that has otherwise no other pathology is an amputating operation that offers no extra benefits when compared to fixing the uterus in its proper position and repairing the pelvic floor system.

## 4. Conclusion

Hysterectomy, whatever the approach used (abdominal, vaginal, laparoscopic), remains the gold standard in the treatment of many uterine benign pathological conditions but we have to encourage the new techniques which use modern technologies and their results are promising and in many cases comparable with hysterectomy.

## Figures and Tables

**Table 1:** Alternative to hysterectomy methods for benign disease.

Laparoscopic adenomyomectomy in case of focal adenomyosis
Isthmical sacropexy (sacrocolpopexy with conservation of the uterus)
Cervicosacropexy after laparoscopic subtotal hysterectomy
Lateral fixation of the cervical stump or the vaginal vault (J.B. Dubuisson method)
Uterine artery embolism (UAE) in case of uterine fibroids
Ttansvaginal temporary uterine artery occlusion
MRI guided focused ultrasound (MRgFUS)
Hysteroscopic (transcervical) resection of the endometrium (endometrectomy) with resectoscope (TCRE)
Hysteroscopic cautery to the endometrium with resectoscope (Rollerball)
Nd-YAG Laser Ablation
Thermal uterine balloon therapy system for endometrial ablation
Global 3D Bipolar Ablation Method
Punctual Vaporizing Method
